# The imperative for universal screening of domestic violence: Social determinants of health disparities during COVID-19 within New Jersey

**DOI:** 10.1016/j.puhip.2025.100597

**Published:** 2025-02-15

**Authors:** Kelly L. Budge, Sameeha Shaikh, Mirai Mikhail, Cassandra Bakus, Sabrina LaRosa, Chinwe Ogedegebe, Antonia F. Oladipo

**Affiliations:** a Hackensack Meridian School of Medicine, Nutley, NJ, 07110, USA; b Hackensack University Medical Center, Department of Emergency Medicine, Hackensack, NJ, 07601, USA; c Hackensack University Medical Center, Department of Obstetrics and Gynecology, Hackensack, NJ, 07601, USA

**Keywords:** Domestic violence, COVID-19, Pandemic, Social determinants of health, Health inequities

## Abstract

**Background:**

It is recognized that stressors encountered during the COVID-19 pandemic created an environment that exacerbated DV. COVID disproportionately impacted at-risk populations, but it is unclear if these social determinants of health disparities similarly impact the incidence of DV. This study aimed to identify and highlight affected communities within the state of New Jersey (NJ) that were disproportionately affected by domestic violence (DV) during the COVID-19 pandemic.

**Study design:**

The study design was retrospective cross-sectional.

**Methods:**

Public data from the NJ Department of Health, Department of Law and Public Safety, and US Census were compared. Community factors, DV incidence, and COVID rates were analyzed using a *t*-test and Spearman correlation.

**Results:**

NJ COVID-19 Incidence rates significantly correlated with varied populations based on socioeconomic status, race, and ethnicity. The median incidence of DV per county population significantly increased from 0.55 % in 2019 to 0.63 % in 2020 (p = 0.03). However, DV incidence was not correlated with rates of COVID-19 per county (p = 0.25). Race and ethnicity did not correlate with DV rates (White, p = 0.06; Black, p = 0.11; 2+ races, 0.14; Hispanic, p = 0.55) except for Asian populations (p = 0.01). Some socioeconomic factors did correlate with DV (unemployment, p = 0.04; median household income, p = 0.003); poverty did not (p = 0.11).

**Conclusion:**

NJ experienced a surge in DV rates during the pandemic that cut across communities of all racial and ethnic backgrounds, in contrast to the more unequal impact of COVID-19 incidence. Findings highlight the importance of screening for DV in times of societal distress to clinicians, researchers, and policymakers.

## 1 Introduction

Domestic violence (DV) is abusive behavior in which one person gains power over another including intimate partner violence (IPV), child abuse, and elder abuse. These types of DV can manifest in forms of sexual, emotional, financial, or psychological abuse. In the United States, over one in three women have experienced rape, physical violence, or stalking by an intimate partner during their lifetime [1]. Similar to IPV, child, and elder abuse are common; with one in seven children and one in ten individuals aged over 60 experiencing abuse in 2021 [2]. The personal and deeply vulnerable experiences of DV survivors often result in fear of speaking out or reporting the abuse, further concealing its true prevalence.

During the COVID-19 pandemic, widespread federal and state mandates severely restricted travel, leading to the closure of non-essential businesses, schools, and workplaces. Unfortunately, this confinement directly and indirectly led to a worldwide increase in DV incidence. Police reports from China's Hubei province documented a threefold increase in IPV rates in February 2020 compared to February 2019 [3]. Similar increases were reported in Argentina, Colombia, Cyprus, Ethiopia, France, Germany, Iran, Kenya, Saudi Arabia, Singapore, Spain, Taiwan, Thailand, the United Kingdom, and Zambia [[3], [4], [5], [6], [7], [8], [9], [10], [11]]. A parallel trend emerged in the United States in Portland, San Antonio, New York City, and Jefferson County, Alabama [3].

COVID-19 disproportionately impacted populations based on their social determinants of health (SDoH) [12]. Considering the increased stressors during the COVID-19 pandemic and the documented increase in DV in the US, we hypothesized that similar SDoH factors that influence COVID-19 incidence would correlate with increased DV incidence. Quantitative insights sought by this study may inform the prioritization of interventions for DV risk factors during a pandemic and support quality improvement initiatives for police departments and pandemic-response agencies.

## 2 Methods

Study design was retrospective cross-sectional. Data acquired were obtained through publicly available resources exempting IRB review. The NJ Department of Health maintains data on COVID-19 infection rates based on mandated reporting. The State of NJ Department of Law and Public Safety releases annual records of the incidences of DV per county (Thirty-Eight Annual Domestic Violence Offense Report 2020). In NJ, law enforcement authorities and crisis organizations collaborate to provide a coordinated response to events of DV, as mandated by N.J.S.A.2C:25-20b(3) (New Jersey Domestic Violence Procedures Manual). The 2020 US Census report reflects a summation of 2016–2020. Additional variables examined included unemployment rate (New Jersey Department of Labor and Workforce), insurance rate (New Jersey State Health Assessment Data), and median household income (United States Census Bureau retrieved from FRED).

Data were analyzed by paired, nonparametric, two-tailed t-tests for two-group comparisons and one-way or two-way ANOVA for multiple independent group comparisons. Nonparametric Spearman correlation was used to compare the linear relationship between variables over time. A p-value <0.05 was regarded as statistically significant. All statistical analyses were performed using Prism, version 10, for Windows (GraphPad Software Inc).

## 3 Results

COVID-19 incidence affected individuals differently based on SDoH disparities. Within NJ, the incidence of COVID-19-positive patients per county was significantly associated with lower socioeconomic status (SES), limited healthcare access, less education, minority populations, and multilingual, multigenerational homes (Table 1). The inverse association of education and COVID-19 was limited to high school degrees and not reflected by higher education. Minority populations, except Asian, were associated with increased incidence of COVID-19.

**Table 1:** Correlations between factors of social determinants of health with the percentage of COVID-positive individuals or DV rates per NJ county. Statistical analysis was performed by Spearman correlation with reported p-values and r-coefficients.

SDoH		COVID-19		DV	
Taxonomy	Factors	p	r	p	r
**Socioeconomic Status**	Poverty	0.0003	0.74	0.1	0.36
	Unemployment	0.01	0.57	0.04	0.45
	Median Household Income	0.009	−0.58	0.003	−0.62
	Median Home Value	0.4	−0.22	0.0001	−0.75
	Median Gross Rent	0.2	−0.32	0.0001	−0.84
	Population Density	0.03	0.51	0.04	−0.46
	Persons per Household	0.1	0.35	0.6	−0.11
**Healthcare Access**	Uninsured (<65y)	0.0009	0.7	0.3	0.29
**Education**	HS Graduate	0.0001	−0.8	0.3	−0.24
	Bachelors or Higher	0.04	−0.48	0.0005	−0.69
**Social and Community Context**	White	0.02	−0.51	0.8	0.06
	Black	0.004	0.63	0.1	0.36
	Asian	0.4	0.23	0.01	−0.55
	Native American	0.01	0.55	0.6	−0.13
	Native Hawaiian or Other Pacific Islander	0.2	0.3	0.7	−0.09
	2+ Races	0.04	0.48	0.1	0.33
	Hispanic	0.003	0.65	0.6	−0.14
	Non-Hispanic	0.003	−0.61	0.5	0.17
	Age 0-5y	0.003	0.74	0.7	−0.1
	Age 6-18y	0.007	0.6	0.9	−0.02
	Age >65y	0.006	−0.61	0.2	0.32
	Disability (<65y)	0.6	0.14	0.0001	0.86
	Language Other than English Spoken at Home	0.01	0.57	0.06	−0.42
	Foreign Born Persons	0.06	0.44	0.01	−0.53
	Veterans	0.05	−0.45	0.002	0.64
	Households with Computers	0.003	−0.65	0.08	−0.39
	Households with Broadband Internet	0.01	−0.56	0.05	−0.43

**Fig. 1:**
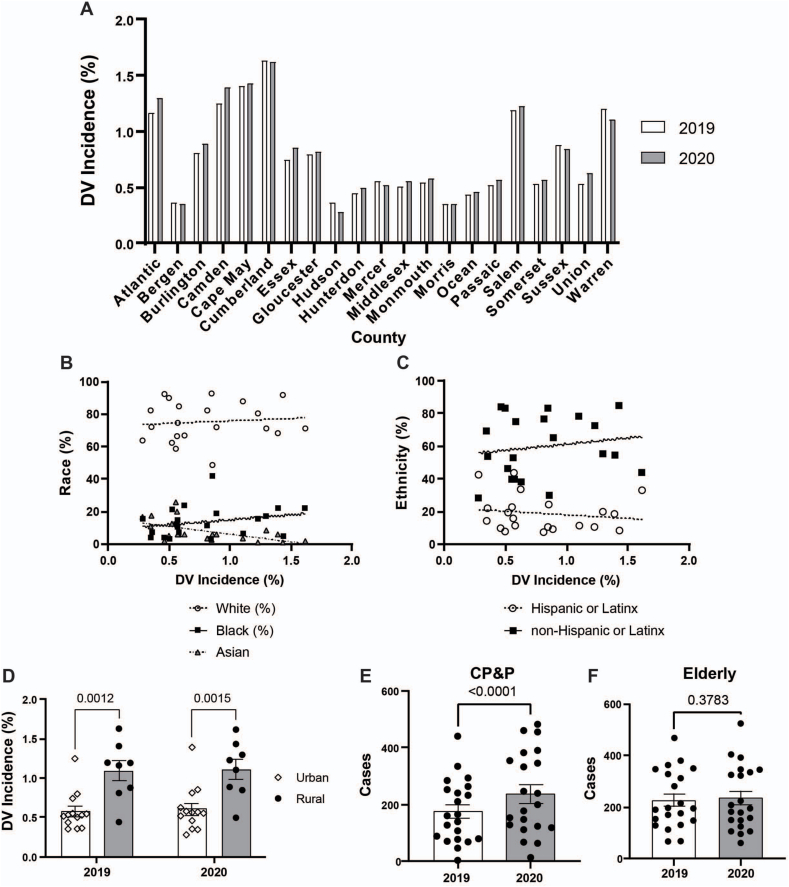
**Domestic violence incidence correlated with social determinants of health factors.** The change in NJ counties' percent of DV incidence per population in 2019 and 2020 (p = 0.032) (**A**). Simple linear regression of DV incidence and percentage of race (**B**) and ethnicity (**C**) per county (White, p = 0.8; Black, p = 0.1; Asian, p = 0.01; Hispanic, p = 0.6; Non-Hispanic, p = 0.5). The incidence of DV per population by population density (**D**). Rural was defined as counties with a population of less than 600 residents per square mile and urban as greater than 900. The number of DV cases involving the state Child Protection and Permanency agency (CP&P) (**E**) and elderly victims greater than 60 years old (**F**) in 2019 and 2020. Each data point represents a single county in NJ. Data were analyzed with paired nonparametric *t*-test, Spearman correlation, 2-way ANOVA with Šidák's multiple comparisons, and Wilcoxon nonparametric *t*-test. A p-value <0.05 was considered significant.

122,693 DV reports were recorded over these two years. Increased rates of DV were observed after the initiation of the COVID-19 pandemic. The median incidence of DV in NJ counties was 0.59 % and 0.64 % in 2019 and 2020, respectively. There was a statistically significant increase in DV rates (p = 0.032, Fig. 1A) which was not associated with rates of COVID-19 per county (R^2^ = 0.086, p = 0.22).

While SES, being disabled, and foreign-born communities influenced the incidence of DV in NJ, healthcare access and minority populations did not (Table 1). Unlike COVID-19, race and ethnicity were not major factors in influencing DV except for populations with a greater percentage of Asian individuals who reported significantly lower rates of DV (Fig. 1B–C). Additionally, rural populations experienced a greater incidence of DV per population than urban counties (Fig. 1D). Poverty and persons per household were not correlated with DV rates compared to other SES factors. While education was a significant factor in DV rates, higher education–not a high school degree–was significantly correlated, indicating that having a Bachelor's degree was protective. Although age did not correlate with the incidence of DV, there was an increased number of calls to child protection and permanency (CP&P) services (p < 0.0001, Fig. 1E–F).

## 4 Conclusion

While there is a robust amount of literature examining relationships between SDoH and COVID-19, to our knowledge, there are no studies examining SDoH and relationships between COVID and DV [12]. Our study showed that this population had a significant correlation between the incidence of COVID-19-positive patients per county and SDoH factors. We found similar correlations between the incidence of DV and factors of SES, education, and social and community context. In contrast to COVID incidence, we found no significant associations of DV with race and ethnicity (except for decreased incidence in Asian race). Our findings reflect known risk factors for DV, which include economic stress (unemployment and low-income [12]), low educational attainment, individuals with disabilities [13], and rural communities [14].

While our data shows differential incidence of DV based on certain characteristics of populations, we emphasize that DV transcends race, ethnicity, and SES and remains persistent among all populations. We emphasize the importance of remaining vigilant in DV screening at all times, but especially during pandemics when healthcare resource availability to the general public is decreased [15]. Effective, compassionate, and equitable screening is necessary to both identify and help victims of DV. Our study underscores the importance of ensuring that all people are receiving equitable screening and the decision to screen patients should not be impacted by race or ethnicity. We urge healthcare workers to stay in accordance with USPSTF and ACOG guidelines on screening for IPV [1,2]. All healthcare providers should be taught how to screen for IPV with tools like HARK, E-HITS, PVS, and WAST [16]; be cognizant of resources in their area and be prepared to offer a private phone line for IPV victims at their clinics and offices. However, screening for child abuse and elder abuse remains more difficult. Experts often recommend multiple tools for screening in these populations due to the lack of a single instrument that contemplates all abuse consequences and forms [17].

Limitations of this study include the problems inherent with retrospective study designs. Due to our reliance on police reports, our collection does not account for survivors who may not have felt safe to report to police, including minority populations of race and sexuality. The conditions of the pandemic may have led to further underreporting due to financial hardships or lack of privacy. We also did not distinguish the subtypes of DV including IPV, elder, and child abuse, nor did we analyze multigenerational homes. Future DV research should highlight vulnerable populations including the LGBTQ + community and determine more effective screening tools for abuse of children, men, and the elderly.

It is also necessary to acknowledge the global burden of domestic violence that varies across cultures, influenced by social norms, economic conditions, and legal frameworks [18,19]. Although our population was limited to a distinct region in North America, we highlight how DV is persistent among all populations. However, addressing this pervasive issue requires culturally sensitive approaches, robust legal protections, and comprehensive support systems to empower survivors and challenge societal norms that perpetuate abuse [20,21].

We emphasize that during periods of calamity, DV persists. The burden of DV is exacerbated by the increased stressors of a pandemic and reduced resources including access to mental health and healthcare. Healthcare workers must be vigilant in screening every patient for DV. We hope that by providing equitable and effective screening, healthcare professionals, researchers, and policymakers can help mitigate the burden of DV.

## What this study adds

● In contrast to the disproportionate effect of the COVID-19 pandemic on marginalized communities, domestic violence cuts across all communities within the state of NJ.

## Implications for policy and practice

● Screening for domestic violence is necessary for all individuals regardless of background.

## Author contribution

KB, CO, and AO were involved in the conception and design of the study. KB, MM, CB, and SL were involved in the acquisition, analysis, and interpretation of data. KB, MM, CB, and SL drafted the article, and CO and AO were involved in revising it critically for important intellectual content. All authors supported the final approval of this version submitted.

## Declaration of funding

None.

## Declaration of competing interest

The authors declare that they have no known competing financial interests or personal relationships that could have appeared to influence the work reported in this paper.
